# Design and Fabrication of Continuous Surface Optical Field Modulator for Angular Spectrum Discreteness Compensation

**DOI:** 10.3390/mi15080952

**Published:** 2024-07-25

**Authors:** Min Xiao, Axiu Cao, Cheng Xu, Hui Pang, Yongqi Fu, Qiling Deng

**Affiliations:** 1 School of Physics, University of Electronic Science and Technology of China, Chengdu 610054, China; 202222120314@std.uestc.edu.cn (M.X.); 201922120310@std.uestc.edu.cn (C.X.); 2 Institute of Optics and Electronics, Chinese Academy of Sciences, Chengdu 610209, China; ph@ioe.ac.cn (H.P.); dengqiling@ioe.ac.cn (Q.D.)

**Keywords:** angular spectrum discreteness, continuous surface profile, optical field modulation, random micro-cylindrical lens array

## Abstract

The light homogenizing element is a crucial component of the illumination system of the lithography machine. Its primary purpose is to realize the uniform distribution of energy. However, it suffers from a common issue, which is angular spectrum discreteness, which significantly impacts light uniformity. To address this, we design and fabricate random micro-cylindrical lens arrays to obtain a small-angle Gaussian optical field, which can compensate for the angular spectrum discreteness. By adjusting the pitches and curvature radii of the micro-cylindrical lenses separately, we are able to manipulate the divergence angle of the emitted sub-beams, enabling precise angular spectrum modulation. By using mask-moving technology, the angular spectrum modulator is fabricated to generate a Gaussian illumination field. The surface profile is measured and determined with a structural roughness below 10 nm. Furthermore, optical test experiments on the modulator have been conducted, achieving an angle error of less than 0.02° and a balance better than 0.5%.

## 1. Introduction

In contemporary technology, the relationship between integrated circuits and lithography machines is inseparable. IntSegrated circuits underpin the digital world, and their performance depends on manufactured accuracy. Lithography machines use highly precise optical systems to project circuit patterns onto semiconductor chips, which affects the performance and density of integrated circuits. The illumination system is one of the essential parts of a lithography machine [1,2,3,4]. It provides uniform illumination on the mask, which directly impacts the resolution of the integrated circuit. High illumination uniformity is an essential requirement for lithography machines to achieve uniform resolution in the exposure field [5,6].

The light homogenizing elements used in lithography illumination systems include microlens arrays (MLAs) [7,8], integrating rods (or homogenizing rods) [9,10], etc. The principle for enhancing beam uniformity is to divide the initial non-uniform light field into smaller segments. By increasing the number of segmented light sources, a higher level of uniformity can be achieved [11,12]. In scalar diffraction theory, the spatial spectrum of the optical field is also called the angular spectrum. By observing the angular spectrum, the intervals among the subdivided light sources indicate the discreteness of the optical field. Thus, the shape of the pupil at the aperture diaphragm appears discretized after the light beam passes through the uniform light element. Overall, angular spectrum discreteness seriously affects the optical field homogeneity [13,14].

To effectively compensate for the discrete angular spectrum, micro-optical elements are used to create a scattering field. This will pad the intervals between the pupils and make the light distribution smoother and more continuous.

In step-and-scan projection lithography systems, dose uniformity across the wafer is crucial as intensity variance affects pattern line width control and, thus, the resolution. Initially, trapezoid illumination was used in lithography illumination systems, but it was later replaced by flat-top Gaussian illumination to further address pulse quantization effects that impact dose uniformity. This can improve energy efficiency and lithography throughput while maintaining fixed slit edges and preventing increased pulse quantization effects [15,16,17]. In practical situations, a small-angle Gaussian optical field is usually used to compensate for angular spectrum discreteness [16,17,18]. The small-angle Gaussian beam produces slight angle changes along the scanning direction, which generates uniform illumination over the chip surface. This improves the resolution and accuracy of lithography.

To generate Gaussian illuminated fields, Deng et al. fabricated a dual-sided concave microlens array, which shows good homogeneity [19]. However, its fabrication is complicated, involving single-pulse femtosecond laser-assisted chemical wet etching. Bitterli et al. proposed concave micro-cylindrical lens arrays to generate Gaussian optical fields by wet etching. The fabrication method is simpler and more convenient [20]; however, the angle control of the modulated optical field is of low accuracy. Ma et al. employed random micro-cylindrical lens arrays to achieve Gaussian beams [21]. Nevertheless, the lens arrays have the same curvature radius with varying pitches, and this causes a minimal sag height of merely 29 nm. This is hardly achievable with state-of-the-art manufacturing technology. Rui et al. proposed using a holographic diffractive optical element (DOE) diffuser to introduce scattering [22], then intervals in the pupil angular spectrum are filled. This makes the uniformity better. In conclusion, both diffractive and refractive optical elements can compensate for angular spectrum discreteness, which contributes to the angular spectrum modulation of the input light.

When DOEs are used to modulate the scattered optical field [23,24,25,26], the uniformity of the light spot is closely related to the number of steps of the element. As the number of steps increases, the diffraction efficiency increases as well. Specifically, when the number of steps is 2, 4, 8, and 16, the diffraction efficiency is 40.5%, 81%, 94.9%, and 98.6%, respectively [27]. But, as the number of steps grows, the fabrication complexity also rises substantially. For example, when the number of steps is 16, for a 193 nm lithography machine, the feature linewidth is hundreds of nanometers, and it is hard to ensure that the alignment error remains within a 50 nm range. This requires high costs and is impractical for large-scale manufacturing. At present, domestic alignment equipment can only achieve an alignment accuracy of 1 μm, which greatly limits the diffraction efficiency of the DOE from reaching its theoretical level and restricts its practical use.

Refractive Optical Elements (ROEs) can achieve precise control of the optical field by dividing and recombining laser wavefronts, enabling functions like beam shaping or homogeneity [28,29,30]. Due to their continuous surface profile, ROEs have high energy utilization efficiency and can be fabricated in one single processso there is no need for multiple alignments. This reduces the complexity and expense of large-scale production. To ensure the feasibility of the paper, the design and fabrication of the angle spectrum modulator are based on ROEs. We propose to modulate the optical field using random micro-cylindrical lens arrays with varying pitches and curvature radii. This not only overcomes the manufacturing constraints but also enables practical applications. Aside from precise control of the light distribution, the light balance is also important. It guarantees the uniformity and consistency of the light distribution and ensures high-quality beam transmission performance in the output pupil plane.

In the paper, we adjust the pitches and curvature radii of the micro-cylindrical lenses separately, which can manipulate the divergence angle of the emitted sub-beams, achieving a Gaussian illumination field. The random micro-cylindrical lens arrays are fabricated by mask-moving technology [31]. In addition, optical test experiments have been conducted to validate its feasibility. The main structure of the article is as follows: the second part introduces the theory of creating a Gaussian optical field through random micro-cylindrical lens arrays, the third part presents the design and simulation of these lens arrays, the fourth part shows the conducted experiments, and the final section gives a summary of the proposed method.

## 2. Theory

**Figure 1:**
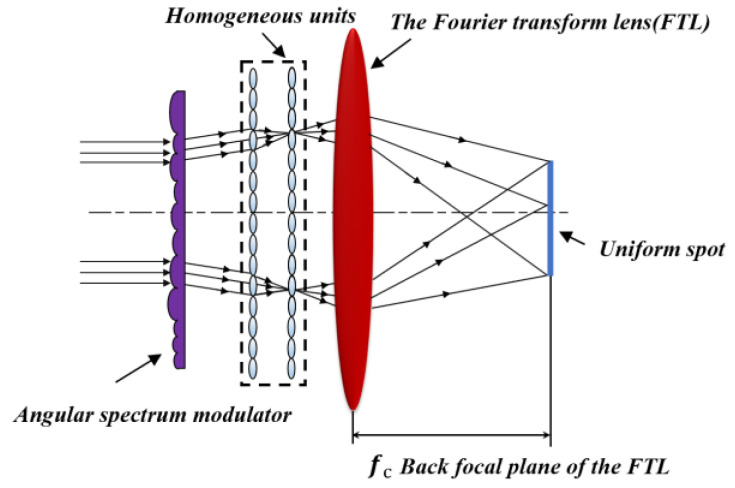
Optical path for angular spectrum discreteness compensation.

To compensate for the angular spectrum discreteness, an angular spectrum modulator needs to be introduced in the lighting system, and its optical path is shown in Figure 1. The modulator consists of multiple sub-cylindrical lens units that have varying pitches, varying sag heights, and are arranged randomly. After the beam passes through the micro-cylindrical lens arrays, the sub-beams exhibit variations in both phase and divergence angles. By controlling the divergence angle of the sub-lenses, a beam satisfying the Gaussian distribution is formed. The beam subsequently traverses the homogeneous units, which divide it into smaller sub-beams. Then, these sub-beams are overlapped in the back focal plane of the FTL by a Fourier lens and produce a uniform spot.

### 2.1. Principles of Structural Parameter Design

The flat-top Gaussian beam is formed by convolving a rectangular function with a Gaussian function. It is a Gaussian distribution in the scanning direction. Thus, the angle spectrum modulator in the paper is a scattering element, which provides Gaussian modulation only in the scanning direction (y-direction), represented by a function $P\left(\theta \right)=e^{\left(-0.5*{\left(\frac{\theta }{\sigma }\right)}^{2}\right)}$, and the modulation angle σ in the paper needs to meet an error range of ±0.02°.

**Figure 2:**
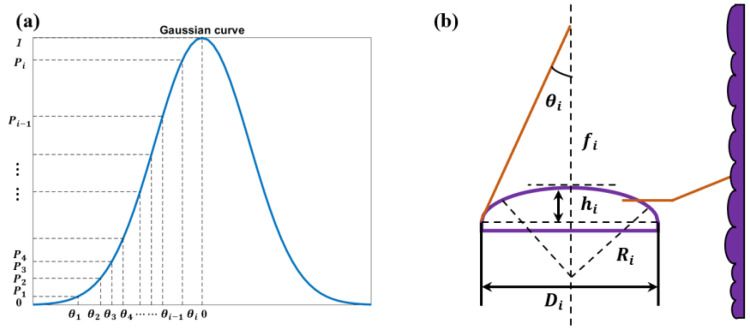
(**a**) Target modulation function; (**b**) micro-cylindrical sub-lens structure.

The approach for realizing Gaussian modulation is universally applicable. Different modulation angles can be achieved based on Figure 2. Figure 2a shows the curve of the Gaussian modulation function. Since both curve and micro-cylindrical lenses are axisymmetric, it is enough to illustrate only half the side along the symmetric curve. The vertical coordinate $P_{i}(i=1\cdots m)$ is the sampled energy distribution, and the horizontal coordinate $\theta_{i}(i=1\cdots m)$ is the corresponding angle. The finer the sampling interval of *P_i_*, the closer the fitted curve is to the designed target. Meanwhile, an accuracy of ±0.02° can be achieved. The structural parameters of the micro-cylindrical lens determine $\theta_{i}$, as presented in Figure 2b. The relationship between $\theta_{i}$, pitch *D_i,_* and focal length *f_i_* is shown in Equation (1).

(1) $$\theta_{i}=\arctan(\frac{D_{i}}{2f_{i}})$$

Equation (2) shows that the sag height is determined by pitch *D_i_* and the focal length *f_i_*.

(2) $$h_{i}=\frac{2f_{i}\left(n-1\right)+\sqrt{4{\left(f_{i}\left(n-1\right)\right)}^{2}-{D_{i}}^{2}}}{2}$$

Here, *n* is the refractive index of the micro-cylindrical lens.

When designing micro-cylindrical lenses, the pitches and focal lengths are adjusted simultaneously to ensure that the sag heights are within the feasible fabrication range.

Assuming the target modulation function is $P\left(\theta \right)=e^{\left(-0.5*{\left(\frac{\theta }{\sigma }\right)}^{2}\right)}$, σ is the modulation angle of Gaussian light, which satisfies σ = 0.66 ± 0.02°. In addition to achieving precise control of the light distribution, it is also critical to ensure the balance of the modulated optical field. This guarantees the uniformity and consistency of the energy distribution. In step-and-scan projection lithography, the balance is usually required to be less than 0.5%. The calculation is shown in Equation (3).

(3) $$Balance_{y}=\frac{\left|E_{+}-E_{-}\right|}{E_{+}+E_{-}}\times 100\%$$

### 2.2. Numerical Simulation of Scattered Optical Field

A structural model is set up to calculate the modulated optical field. Specifically, the diffracted far-field distribution is obtained by Fourier transform. The parallel beam is modulated by the angular spectrum modulator, assuming that the complex amplitude of the modulator is g(x,y). Based on the scalar diffraction theory, the far-field diffraction distribution $G\left(f_{x},f_{y}\right)$ is acquired by a two-dimensional Fourier transform [32], shown in Equation (4).

(4) $$G\left(f_{x},f_{y}\right)=\iint g\left(x,y\right)e^{-i2\pi (f_{x}x+f_{y}y)}dxdy$$

In the Equation, $f_{x}$, $f_{y}$ represents the spatial frequency. The relationship between physical space and frequency space coordinates is shown in Equation (5).

(5) $$f_{x}=\frac{x}{\lambda z},f_{y}=\frac{y}{\lambda z}$$

Thus, we obtain that Equation (4) is applicable to the entire spectrum, but it depends on the wavelength.

According to Fourier optics, a phase shift in the spatial domain causes location movement in the frequency domain, as shown in Equation (6).

(6) $$F\left\{g\left(x-x_{0}\right)\right\}=e^{-j2\pi \xi x_{0}}G\left(\xi \right)$$

**Figure 3:**
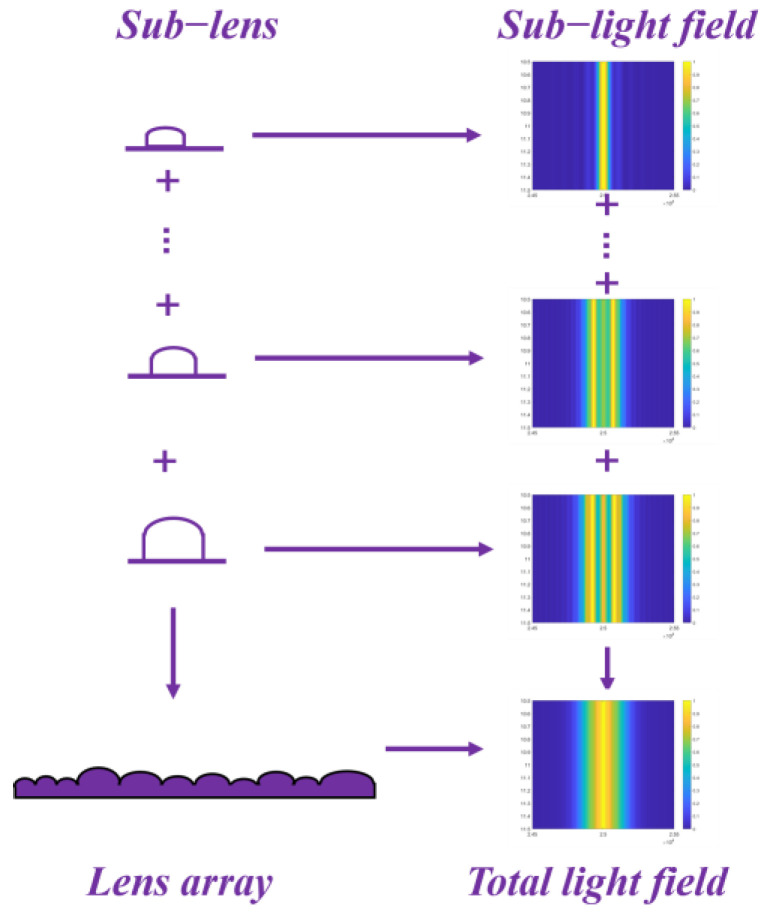
The formation of Gaussian light field.

When the sub-lenses are arranged in the spatial domain, the sub-light fields generated are superimposed in the frequency domain, as shown in Figure 3. The pitch *D_i_* and focal length *f_i_* of the sub-lens will affect the generated angle of the sub-lens, thus influencing the diffracted light fields. Each sub-light field is a Gaussian distribution, superimposed on each other to form the final target Gaussian light field.

## 3. Design and Simulation

**Figure 4:**
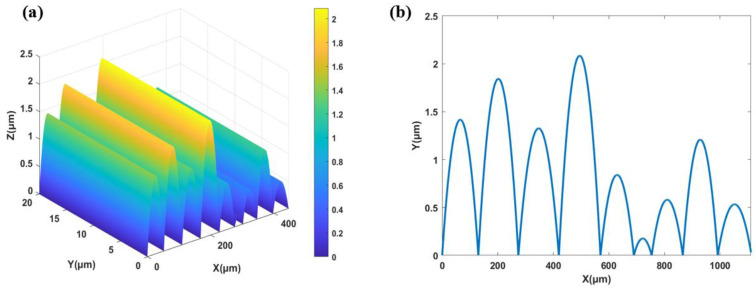
Structural profile with single cycle: (**a**) three-dimensional morphology; (**b**) one-dimensional profiles.

According to the design theory, the target Gaussian function curve is sampled. During the design process, *P_i_* is sampled at 9 points, denoted as P_1_ to P_9_. Thus, the corresponding angle $\theta_{1}$ to $\theta_{9}$ can be obtained, and their corresponding energy values are listed in Table 1. Based on $\theta_{1}$ to $\theta_{9}$, we have designed random micro-cylindrical lenses with pitches ranging from 64 μm to 149.5 μm and curvature radii ranging from 1341.1 μm to 3463.6 μm. The resulting sag heights range from 173 nm to 2083.2 nm, which is within the feasible fabrication range. Specific parameters can be found in Table 1. Then, multiple periods are used to cover the entire aperture, forming a complete structure. The structure within a single period is shown in Figure 4, where Figure 4a represents the three-dimensional morphology, and Figure 4b shows the one-dimensional profile. The length of a period is 1112.1 μm. During the design and testing, visible light with a wavelength of 532 nm is used as the light source.

**Figure 5:**
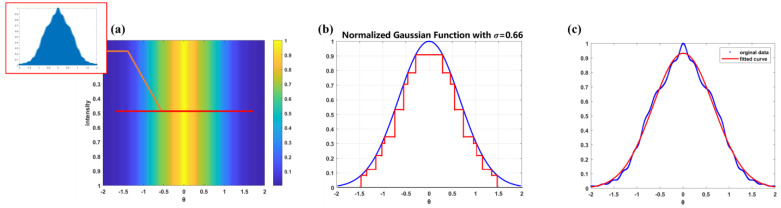
(**a**) Far−field scattered optical field; (**b**) superposition of rectangularly illuminated optical fields; (**c**) original curve and the fitted Gaussian distribution.

The far-field distribution of parallel light passing through the modulation device is shown in Figure 5a, which is a single-direction Gaussian light. In fact, the light field is a superposition of rectangular optical fields, as shown in Figure 5b. By normalizing the gray values of the far-field optical field, we can obtain the fitted Gaussian curve. Assuming the diffraction distance is *z* and the incident light spot size is *S*, then the sampling interval on the diffraction plane is determined by dx’=λ⋅z/S. If the pixel number of the diffracted light spot is *M*, the scattering angle is calculated by tanθi=dx’⋅M/z, which is tanθi=λ⋅M/S. Then, a fitted Gaussian function can be obtained. By analyzing the function, we can calculate the corresponding modulation angle σ when the energy attenuates to $1/\sqrt{e}$. The final fitted angle spectrum modulation function is $P’\left(\theta \right)=e^{\left(-0.5*{\left(\frac{\theta }{\sigma ’}\right)}^{2}\right)}$, σ’=0.66, as shown in Figure 5c. Additionally, using Equation (1), the balance is 0.46%.

## 4. Experiments and Discussion

### 4.1. Manufacturing Procedure

**Figure 6:**
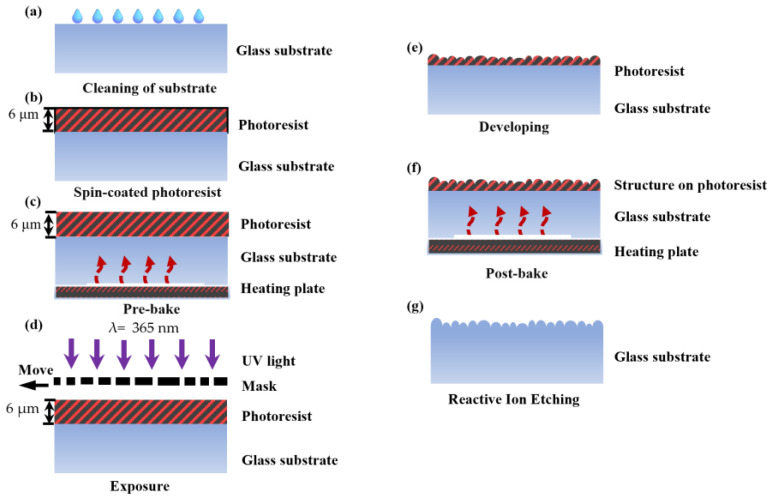
The fabrication process of random micro-cylindrical lens arrays: (**a**) cleaning of the substrate; (**b**) spin-coated photoresist; (**c**) pre-bake; (**d**) exposure; (**e**) developing; (**f**) post-bake; (**g**) reactive ion etching.

The angular spectrum modulator is a continuous faceted structure, and its fabrication is based on mask-moving technology [33]. During the fabrication, we study the nonlinear relationship between the exposure amount and exposure depth and control the exposure time and other processing parameters. Thus, its structure on the photoresist molding can be achieved, and a mature surface-forming system is formed. The specific process is shown in Figure 6, and the specific parameters are shown in Table 2.

First, as seen in Figure 6a, the substrate is cleaned to make sure it is dust-free and oil-free. The cleaning is usually completed with a chemical solvent, such as an organic solvent cleaning solution, alkaline, acidic cleaning solution, etc. The concentration of acidic cleaning solution is generally controlled at 15% to 20%. After that, the base surface is left to dry. Afterwards, the photoresist is spin-coated on the surface of the substrate using a spin-coater, as shown in Figure 6b. The rotational speed and coating time of the spin-coater are precisely controlled to ensure a consistent and uniform coating. The type of the photoresist is 10XT. The relevant parameters include a rotational speed of 4000 rpm/min, a time of 40 s, and a viscosity of 520 CP. Then, as depicted in Figure 6c, a pre-bake process is conducted, where the substrate is dried on a hot plate at 100 °C for 5 min. After the pre-bake, the thickness of the photoresist is assessed by a step profilometer (Stylus Profiler System, Dektak XT, Bruker, Karlsruhe, Germany) with the measured value of 6 µm. Subsequently, as illustrated in Figure 6d, the mask is aligned with the substrate, and mask-moving technology is executed to expose the desired pattern. The exposure power density is adjusted to 3.5 mW/cm^2^, and the center wavelength of the light source is 365 nm. During the exposure process, the mask is moved at a distance of 10 µm, and the pre-exposure time is 5 s. Next, as shown in Figure 6e, the exposed substrate is placed into a diluted developer solution, where the concentration ratio of the developer solution is AZ400K:Deionized H_2_O = 2:3, with a duration of 25 s. Subsequently, as depicted in Figure 6f, a post-bake process is conducted to cure the photoresist at 110 °C for 30 min. At last, as shown in Figure 6g, the exposed substrate is etched to create the desired micro-nanostructures using specialized etching equipment. The etching process involved using a gas mixture of SF_6_ and CHF_3_ in a ratio of 23:33, with an etching time of 5000 s and pressure of 1.5 Pa. The whole process needs to strictly control the parameters of each step to ensure the final preparation of a high-quality random micro-cylindrical lens array.

**Figure 7:**
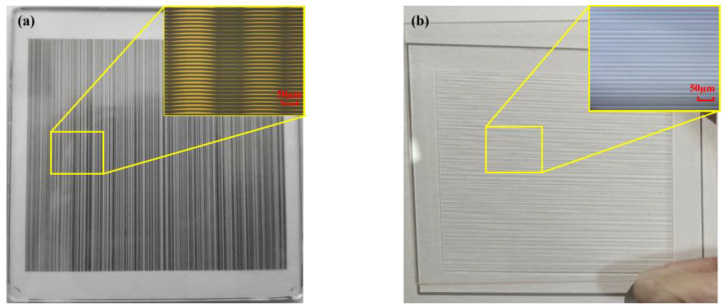
Schematic of processing structure: (**a**) binary mask; (**b**) random micro-cylindrical lens arrays.

As shown in Figure 7a, a binary mask is fabricated based on the target structure. Accordingly, random micro-cylindrical lens arrays are fabricated, as shown in Figure 7b, with sag heights ranging from 167 nm to 2083 nm and pitches from 64 μm to 149.5 μm.

### 4.2. Measurement and Characterization

**Figure 8:**
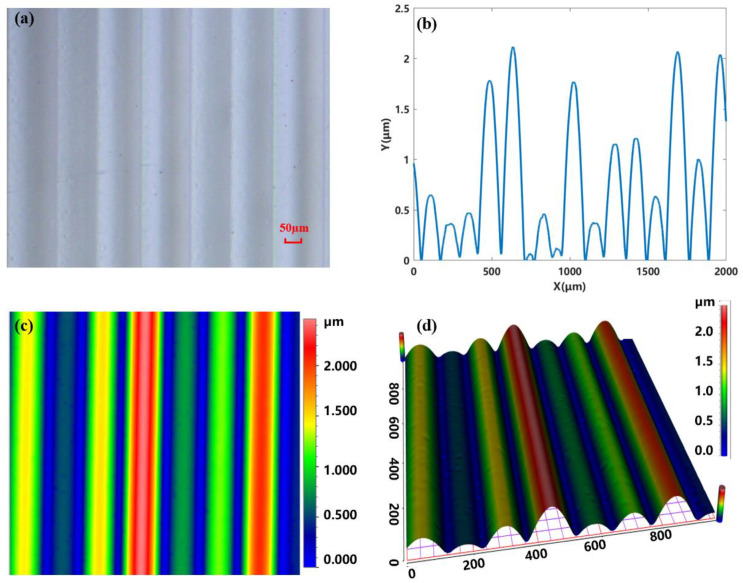
(**a**–**d**) show the structural morphology under different observation means.

To comprehensively evaluate the performance of the structure, we use a set of precise measurement and characterization techniques. A microscope (Olympus BX51, Olympus, Tokyo, Japan) is used to obtain microscopic pictures, as shown in Figure 8a. It can be seen that the structure of random micro-cylindrical lens arrays is smooth, and all sub-lenses are rectangular in shape. As presented in Figure 8b, the pitches and sag heights of the micro-cylindrical lens are identified by a step profilometer (Stylus Profiler System, Dektak XT, Bruker, Karlsruhe, Germany). The maximum and minimum pitch are measured to be 150.1 µm and 64.1 µm, corresponding to the deepest and shallowest sag heights of 1920 nm and 101 nm, respectively. To describe the surface morphology, an optical three-dimensional (3D) surface profiler (SuperView W1, CHOTEST, Shenzhen, China) is used. The two-dimensional (2D) profile of the processed structure is shown in Figure 8c, and the 3D sketch is shown in Figure 8d. We can see that the 2D profile has continuous lines, and its three-dimensional morphology has good geometric features and smoothness. The surface roughness is also assessed by the step profilometer. During the measuring process, four areas of the substrate are chosen to quantify roughness. The measured Root Mean Square (RMS) roughness for the top, waist, bottom, and back are 1.97 nm, 9.8 nm, 6.0 nm, and 1.3 nm, respectively. This reveals that random micro-cylindrical lens arrays can be constructed by mask-moving technology with a smooth surface and great graphical fidelity.

### 4.3. Optical Experiments and Discussion

**Figure 9:**
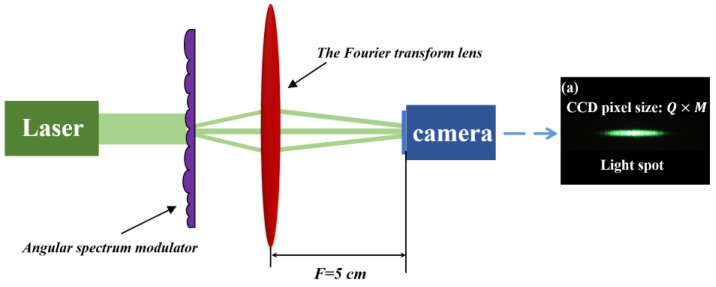
Test optical path: (**a**) Modulated light distribution.

The optical performance of the prepared structure is tested and characterized below. The test optical path is shown in Figure 9. The 532 nm light source is modulated by an angular spectrum modulator and transformed by a Fourier lens. Then, the modulated optical field is collected at the focal plane of the Fourier lens with a camera (HR16000CTLGEC, SVS-VISTEK, Seefeld, Germany). The modulated light distribution is shown in Figure 9a.

**Figure 10:**
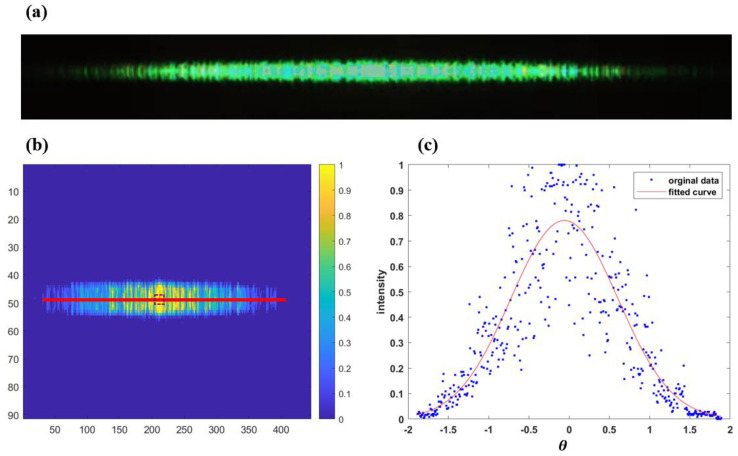
(**a**) light spots under 532 nm light source; (**b**) selection of the row of light spot centroid; (**c**) Gaussian fit of the centroid row.

Further, MATLAB R2022b is used to analyze the data of the collected light spot. The light spot under the 532 nm light source is shown in Figure 10a. We choose the row of centroid, as shown in the red curve in Figure 10b. From the curve, the data points are shown in blue dots in Figure 10b, and the Gaussian fitted curve is the red curve in Figure 10c. Using the CCD pixel size Q (7.4 μm), the number of pixels M with different energy, and the detection distance F (5 cm), we can calculate the angle using the geometrical relationship tanθy=Q⋅M/F. This allows us to obtain the angle σ under the fitted curve. he final modulation angle σ is 0.6655 and the balance is 0.2%, which satisfies the index of balance <0.5%.

**Figure 11:**
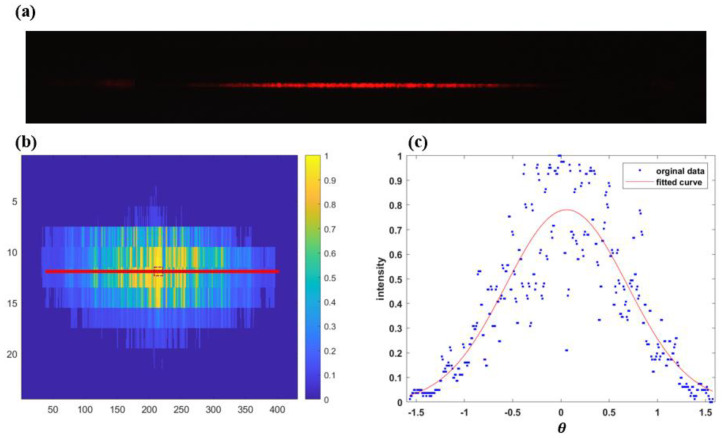
(**a**) light spot under 650 nm light source; (**b**) selection of the row of light spot centroid; (**c**) gaussian fit of the centroid row.

To further verify the performance of the angle spectrum modulator, we conduct experiments at a wavelength of 650 nm. The corresponding modulation angle *σ* is 0.63°. The realistic light spot is shown in Figure 11a, and the centroid row is shown in Figure 11b. With the data of the row, the Gaussian fitted curve is shown in Figure 11c. The final modulation angle *σ* in 650 nm wavelength is calculated as 0.6295, with a balance of 0.4%, meeting the requirements of an error range of ±0.02° and balance <0.5%. This proves that the device has a high fitting accuracy.

## 5. Conclusions

In this paper, we propose the design of novel random micro-cylindrical lens arrays to achieve compensation for angular spectrum discreteness. By adjusting the pitches and curvature radii of the micro-cylindrical lenses separately, we are able to manipulate the divergence angle of the emitted sub-beams, allowing precise angular spectrum modulation. Using mask-moving technology, the angular spectrum modulator is fabricated to generate a Gaussian illumination field. The surface profile is measured and determined with a structural roughness below 10 nm. Furthermore, optical test experiments on the modulator are conducted, achieving an angle error of less than 0.02° and a balance better than 0.5%.

## Figures and Tables

**Table 1:** Parameters of the sub-lenses.

i	1	2	3	4	5	6	7	8	9
$P_{i}$	0.08	0.12	0.22	0.30	0.34	0.53	0.71	0.78	0.91
$\theta_{i}/{}^{\circ}$	1.47	1.35	1.15	1.02	0.96	0.74	0.55	0.46	0.29
$D_{i}/\mu m$	149.5	144	130	125.4	145.6	120	112	121.6	64
$R_{i}/\mu m$	1341.1	1407	1491.3	1627.6	1998.9	2141.6	2700	3463.6	2900
$h_{i}/nm$	2083.2	1842	1416.5	1325.7	1325.7	840	580	533.6	176

**Table 2:** Lithography process and parameters.

Lithography Process	Parameter
Cleaning of substrate	Chemical solution cleaning, drying
Spin-coated photoresist	10XT, 4000 rpm/min, 40 s
Pre-bake	100 °C, 5 min, 6 µm
Exposure	λ = 365 nm, 3.5 mW/cm^2^, pre-exposure 5 s
Developing	AZ400K:Deionized H_2_O = 2:3, 25 s
Post-bake	110 °C, 30 min
Reactive ion etching	SF_6_:CHF_3_ = 23:33, t = 5000 s, *P* = 1.5 Pa

## Data Availability

Data will be provided on request due to privacy through the corresponding author (Axiu Cao) of this article.
